# Morphometric Wings Similarity among Sylvatic and Domestic Populations of Triatoma infestans *(Hemiptera: Reduviidae) from the Gran Chaco Region of Paraguay*

**DOI:** 10.4269/ajtmh.16-1013

**Published:** 2017-05-30

**Authors:** Antonieta Rojas de Arias, Ana Laura Carbajal de la Fuente, Ana Gómez, María Carla Cecere, Miriam Rolón, María Celeste Vega Gómez, Cesia Villalba

**Affiliations:** 1 Centro para el Desarrollo de la Investigación Científica (CEDIC), Diaz Gill Medicina Laboratorial/Fundación Moisés Bertoni, Asunción, Paraguay; 2 Laboratorio de Eco-Epidemiología, Instituto de Ecología, Genética y Evolución de Buenos Aires (IEGEBA), Consejo Nacional de Investigaciones Científicas y Técnicas (CONICET), Buenos Aires, Argentina; 3 Laboratorio de Eco-Epidemiología, Departamento de Ecología, Genética y Evolución, Facultad de Ciencias Exactas y Naturales (FCEyN), Universidad de Buenos Aires (UBA), Buenos Aires, Argentina; 4 Programa Nacional de Control de la Enfermedad de Chagas, SENEPA, Asunción, Paraguay

## Abstract

Despite sustained efforts for eliminating *Triatoma infestans*, reinfestation still persists in large part of the endemic area of Chagas disease from the Gran Chaco region. Sylvatic *T. infestans* populations seem to threat success of control programs of domestic *T. infestans*. In this study, we analyze whether *T. infestans* collected after a community-wide spraying were survivors or were immigrants from elsewhere using geometric morphometric tools. We used 101 right wings of female *T. infestans* captured before and after intervention program carried out in 12 de Junio and Casuarina, villages from Paraguayan Chaco, and in Puerto Casado during presprayed collection. There were no significant differences in wing size of domestic *T. infestans* between pre- and postspraying populations, and between domestic and sylvatic ones. When shape variables originating from postintervention individuals from 12 de Junio were introduced one by one into a discriminant analysis, the greatest weight (53%) was allocated to the sylvatic group. Furthermore, from the prespraying population, 25% were reallocated as postintervention individuals. Only 11% of the insects were reassigned to other groups Puerto Casado and Casuarina. These results suggest that postspraying individuals appear to have different origins. Half of the postspraying individuals from 12 de Junio were similar to the sylvatic ones and 25% of these were similar to those captured in the prespraying period. This remarkable morphometric wings similarity between sylvatic and domestic populations is new evidence suggesting that they could be highly related to each other in the Paraguayan Chaco; human-fed bugs from sylvatic area also support this.

## Introduction

The Gran Chaco eco-region and adjacent areas extending from Argentina, Bolivia, and Paraguay are highly endemic for Chagas disease. Despite the current campaigns for the elimination of the major vector *Triatoma infestans* (Hemiptera: Reduviidae), transmission and reinfestation persist in wide areas of this region.^1^ This fact interrupts the success in eliminating vectorial transmission because control programs have been conducted against domestic populations of *T. infestans* using residual insecticide spraying of human dwellings,^2^ and did not involve the potential reinfestation process from sylvatic areas.^3^^,^^4^ Therefore, this new epidemiological scenario is a priority issue for the Southern Cone Initiative.^1^^,^^5^

In the last decades, the sylvatic populations of *T. infestans* have been reported in Bolivia,^6^^–^^8^ Argentina,^9^ Chile,^10^^,^^11^ and Paraguay.^12^^,^^13^ These records have challenged the hypothesis that this vector is strictly domestic and peridomestic. Recent studies have shown that remnant triatomine populations exist in intradomiciles after spraying campaigns in endemic communities are highly connected to sylvatic colonies and they could be involved in restoring the reinfestation process.^14^ To support this new paradigm, recent genetic studies in the Andes showed that first-generation migrants within different populations have provided evidences of insect movement from the sylvatic to the intra- and peridomestic areas, enhancing the hypothesis of vector transmission risk from the invasion of human habitats by sylvatic populations of *T. infestans*.^15^^,^^16^ These data show the relevance of sylvatic *T. infestans* populations in their possible role in the recolonization of treated areas and potential exchange between sylvatic and intraperidomestic triatomine populations in a wide geographical area of Grand Chaco under epidemiological risk of transmission of *Trypanosoma cruzi.*

Moreover, other several sources of reinfestation by *T. infestans* were identified after house-insecticide spraying in the Gran Chaco region. In Chuquisaca, Bolivian Chaco, a molecular genetic study suggests that triatomine populations are probably residues coming from peridomestic habitats and they are characterized with an active dispersal ability and migration.^17^ In the dry and humid Argentinean Chaco, potential sources of reinfestation such as external foci (neighboring localities or unknown foci) and residual foci (persistent bug populations surviving insecticide application or insecticide resistant bugs) were also identified.^18^^–^^23^ Furthermore, in other areas where sylvatic *T. infestans* foci were potentially discarded, reinfesting populations of *T. infestans*, which were determined by quantitative morphology, corresponded to mixed survivor populations drift from treated areas and neighboring habitats, whose phenotype may have originated from genetic derivation or selection caused by the insecticide.^24^

In the Paraguayan Chaco, rapid reinfestation of triatomine populations was observed in indigenous dwellings.^25^^,^^26^ Previous genetic studies on sylvatic samples have shown similar haplotypes in sylvatic and domiciled individuals of *T. infestans* captured in the Paraguayan Central Chaco.^13^ However, it was unknown if these populations were survivors from residual spraying, control failure, pyrethroid resistant, or if they came from external reinfestation. As part of a longitudinal research in rural areas of the Paraguayan Chaco, we investigated the house infestation with *T. infestans* in a well-defined study area and analyze the potential origin of the *T. infestans* reinfestation in two rural localities after insecticide spraying using the geometric morphometry of triatomine wings. We tested if the *T. infestans* captured in postspraying periods exhibits close similarities with those captured during prespraying in the same locality, in the closest neighbor locality, in the sylvatic environment near to them, and in a locality with high inhabitants interchange from where they could have arrived by passive transportation. Sources of feeding and *T. cruzi* infection analysis of triatomines were considered for assessing the potential risks of vector-borne transmission after spraying.

## Materials and Methods

### Study sites.

**Figure 1.**
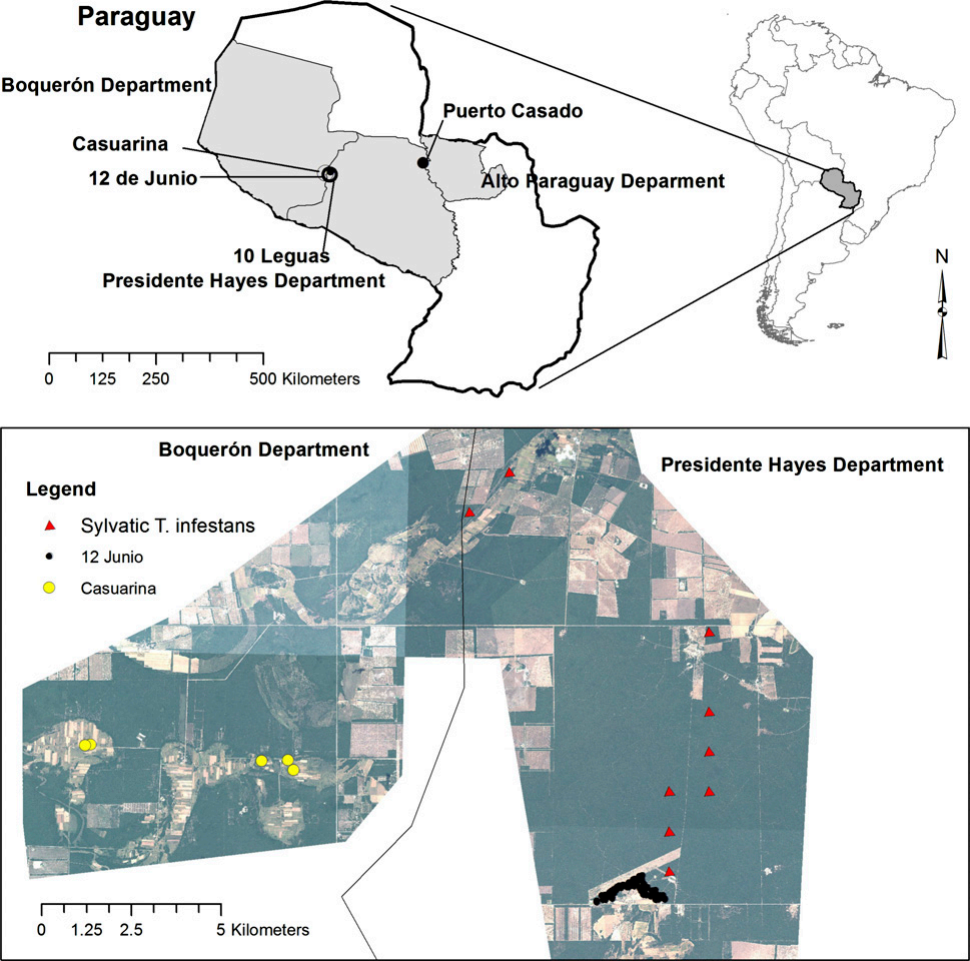
The fieldwork was carried out in 12 de Junio and Casuarina, two indigenous communities of Central Chaco in Paraguay. This figure appears in color at www.ajtmh.org.

The fieldwork was carried out in 12 de Junio and Casuarina, two indigenous communities of Central Chaco in Paraguay (Figure 1). The area corresponds to xeromorphic woods of the Gran Chaco eco-region with some characteristic species such as *Aspidosperma quebracho-blanco*, *Schinopsis balansae*, *Bulnesia sarmientoi*, *Prosopis nigra*, *Calycophyllum multiflorum*, and *Stetsonia coryne* sp.^27^^,^^28^ The climate in Chaco Central is characterized by extreme heat in the summer and mild temperatures in the winter. High temperatures have been registered to reach 45°C in spring and summer times and low temperatures reached −7°C in winter times. Wind blows at an average speed of approximately 11.9 km/h that increases up to 14.0 km/h in the winter.^29^

The villages of 12 de Junio and Casuarina are located in Presidente Hayes Department and are distributed over approximately 500 km^2^; they have 570 multiethnic inhabitants and the distance between villages is around 15 km. A total of 111 dwellings are distributed between in 12 de Junio (*N* = 60) and Casuarina (*N* = 51) villages. The average number of people per household in the village of 12 de Junio is 6.7, whereas the average year of schooling is 0.6. The origin of this ethnic group is unclear but Fabre (2005) divides them into two groups. The first lived in the Chaco bank of the Paraguay River in Puerto Casado (now Puerto Victoria) and the second group moved into the area of the Mennonite farms. Inhabitants of 12 de Junio correspond to a group of “Angaité” who originally lived in the area of Puerto Casado, Alto Paraguay Department (bordering the eastern region). The average number of people per household in the Casuarina village is 5.2, whereas the average year of schooling is 2.9. Most people live in dwellings with trunk wall and mud-brick walls without plaster (French Wall), mud or mixtures of both materials. Roofs are mostly from zinc in an attempt to solve the problem of water shortage of the region.

### Entomological surveys.

An entomological survey for searching domestic triatomines was conducted in March 2008, before residual insecticide spraying campaign (prespraying period). Then an entomological follow-up was conducted at 3, 6, 9, and 12 months postspraying in 12 de Junio and Casuarina. The baseline entomological survey included searches for triatomine bugs in domestic and peridomestic habitats if any, in all the dwellings of the study area. In a total of 111 dwellings, two trained collectors searched for triatomine bugs in bedrooms and peridomestic areas during 30 minutes per house. The triatomine bugs collected were placed in plastic glasses identified with the name of the household head, house number, and specific site of collection. Dwellings were sprayed with lambda cyhalothrin (10% wettable powder) at 30 mg/m^2^ using manual compression sprayers (X Pert Hudson^®^, Chicago, IL) by experimented field workers from the Chagas Disease National Control Program. Immediately after insecticide spraying, knocked down triatomines were collected, as well. A self-sealing plastic bag was given to each family for placing any triatomine bug captured in the domestic or peridomestic areas. The intradomiciliary captures were carried out in all months following the same procedure previously explained. House infestation pertains to the finding of at least one *T. infestans* in domestic or peridomestic sites.

The collection of sylvatic *T. infestans* was carried out five times by 2 days each over the months of May to August 2010 in areas around the road between 12 Junio and 10 Leguas, which are separated 8 km from each other as described in Rolón and others (2011).^13^ Sylvatic triatomines were captured on dry branches and nests of birds, using manual revision of demarcated areas throughout the day with the help of Nero, a 9-month-old gray German shepherd dog.^13^ The 10 Leguas village was simultaneously intervened with a blanked spraying campaign as well as 12 de Junio; however, reinfestations or adult captures were not detected in their human dwellings in any of the months of postspraying and few adults were captured during the baseline, then was not included in the sample.

### Morphometric geometric of wings.

We used females *T. infestans* collected during the pre- (baseline) and postspraying periods (3, 9, and 12 months) due to the low number of males. Pre- and postspraying collected sites (with at least 10 intradomiciliary *T. infestans*) from 12 de Junio and Casuarina localities were included. A sample of intradomiciliary *T. infestans* from Puerto Casado collected in 2005 was incorporated to analysis due to the potential original settlement of 12 Junio inhabitants. Sylvatic triatomines, collected in 2010 from the neighborhood of 12 de Junio, were incorporated to the analysis.

**Figure 2.**
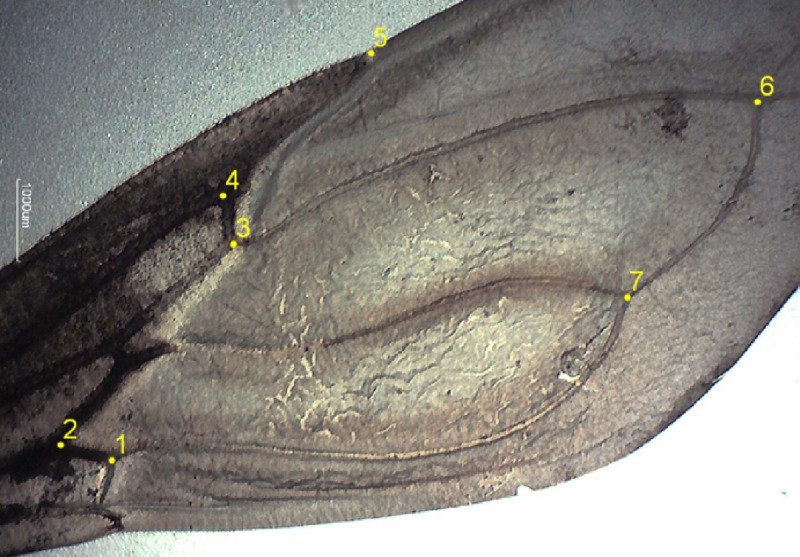
Digitalized landmarks of right female wings of *Triatoma infestans* from Paraguayan Chaco. This figure appears in color at www.ajtmh.org.

A total of 101 wings from females of *T. infestans* were removed with forceps and mounted between microscope slides and coverslips using a commercial adhesive. Images were obtained using a digital camera (Moticam 1000 1.3 MP Live Resolution), using software Motic Image Plus 2.0 (Kowloon, Hong Kong, China) connected to a stereoscopic microscope (Quimis 08021363, model Q714Z-2; Diadema, Sao Paulo, Brazil). A total of seven “type I” landmarks (venation intersections, according to Bookstein, 1991)^30^ were selected (Figure 2). We did not use all possible landmarks due to the limitation of having few specimens mainly in the smallest group.^31^ Only right wings were included in the analyses to avoid pseudoreplication.

To compare the overall wing size between populations of *T. infestans*, we used the isometric estimator known as “centroid size” derived from coordinate data. Centroid size is defined as the square root of the sum of the squared distances between the center of the configuration of landmarks and each individual landmark.^31^ Shape variables as described by Bookstein (1990) were obtained using the Generalized Procrustes analysis superimposition algorithm.^32^ The resulting variables are called partial warps (PWs).^33^ The method is based on the superimposition of each individual using least-square criterion, eliminating effects of scale, orientation, and position of the objects. The shape variables define the positional changes at each landmark in relation to a consensus shape.

### Statistical analysis.

Landmark repeatability was tested in 100% of wings, digitized twice by the same person. The measurement error was estimated by the “repeatability” index (*R*) as described by Arnqvist and Martensson (1998),^34^ which can be regarded as the Pearson correlation coefficient between two measurements. Kruskal–Wallis tests corrected by Bonferroni's method were used to analyze the isometric size. The shape proximity was analyzed using Mahalanobis distances.^35^ These were derived from shape variables and their statistical significance was computed by permutation tests (1,000 runs each) after Bonferroni correction. The distances were used in an unweighted pair-group method with arithmetic average (UPGMA) cluster analysis to produce a dendrogram. To detect allometry (i.e., to establish whether variation in shape was affected by variation in size), the shape variables were regressed on the centroid size by multivariate regression analysis.

Postspraying specimens from 12 de Junio were entered one by one to the discriminant analysis of prespraying and sylvatic samples (reference group) and assigned to these reference groups with which they had the shortest Mahalanobis distance. Each individual classification was performed on shape variables computed from the total of reference specimens plus the individual to be classified; thus, for each classification, shape variables of the reference specimens were recomputed after adding only one postintervention individual as proposed by Dujardin and others (2010) and Gaspe and others (2013).^20^^,^^36^ The percentage of postintervention specimens assigned to each reference group was computed.

The geometric coordinates of each landmark were digitized using *tpsDig2*, version 2.09 (free software developed by Rohlf, available at www.life.bio.sunysb/morpho). *VAR* was used for the precision test in the digitization of landmarks and for the nonparametric comparisons of centroid size, *MOG* for Procrustes superimposition, generation of PW, assignment of “unknown specimens,” and validated reclassification tests; *PAD* for computing the Mahalanobis distances; *COV* for examination of residual allometry within shape variables. The UPGMA dendogram was obtained using Phylip, version 3.6 (Seattle, WA),^37^ through *PAD*. The modules *VAR*, *MOG*, *PAD*, and *COV* developed by J. P. Dujardin are included in the *CLIC* package (free software package available at www.mome-clic.com).

### Infection with *T. cruzi* and blood meal sources.

Infection with *T. cruzi* was determined by microscopic observation of feces at 400× magnification for all *T. infestans* captured alive.

Blood meal contents were examined on 49 sylvatic *T. infestans* collected in the neighborhood of 12 Junio and 10 Leguas, and 12 intradomicile *T. infestans* from 12 de Junio and 10 Leguas (*N* = 9). All triatomines were dissected, coded, frozen, and stored at Centro para el Desarrollo de la Investigación Científica, Paraguay. A random sample of 30 (61%) sylvatic and a total 87 (100%) intradomiciliary bugs were tested against human, dog, chicken, cat, and goat antisera. Antisera from wild animals were not included in these assays because they were not available commercially. A direct enzyme-linked immunosorbent assay (ELISA) was used to identify blood meal sources of triatomines.^38^

## Results

### House reinfestation by *T. infestans*.

Baseline dwelling infestation by *T. infestans* in 12 de Junio of 70% (42 houses) and Casuarina 43.1% (22 houses) dropped sharply in both localities up to the first-month postspraying (0%) and remained with 1.6% and 11.8% until the 6-month follow-up, respectively. The locality of 12 de Junio did not present peridomestic habitats and Casuarina showed 5.9% (3/51) of peridomestic infestation. All infested dwellings after the insecticide spraying were previously infested at baseline with *T. infestans* (Figure 1). Infection with *T. cruzi* was not detected in any of the evaluated triatomine bugs.

### Morphometric analyses.

Comparison of two digitization sets for the same specimens showed good repeatability for centroid size (*R* = 0.99) and the relative warps (*R* = 0.93).

**Figure 3.**
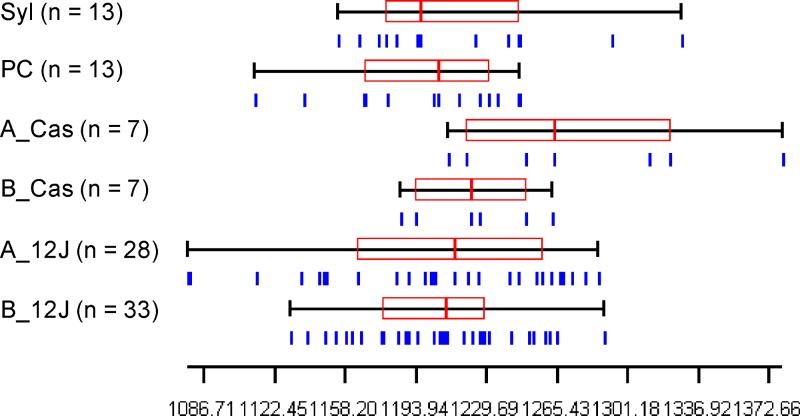
Wing centroid size distribution for *Triatoma infestans* females collected before (B) and after (A) pyrethroid spraying at “12 de Junio” (12J), “Casuarina” (Cas), and “Puerto Casado” (PC) localities in Paraguayan Chaco. Sylvatic (Syl) bugs were captured next to 12J. Vertical lines (blue) under the quantiles represent specimens. Each box denotes the median as a line across the middle and the quartiles (25th and 75th percentiles) at its ends. This figure appears in color at www.ajtmh.org.

Kruskal–Wallis tests give no significant values (*P* > 0.01) for comparisons of wings size between pre- and postspraying pairs from each population of *T. infestans*. Similarly, there were no significant differences in the size of the wings of sylvatic individuals when compared with prepopulations and/or postspraying ones (Kruskal–Wallis test, *P* > 0.01) (Figure 3).

**Figure 4.**
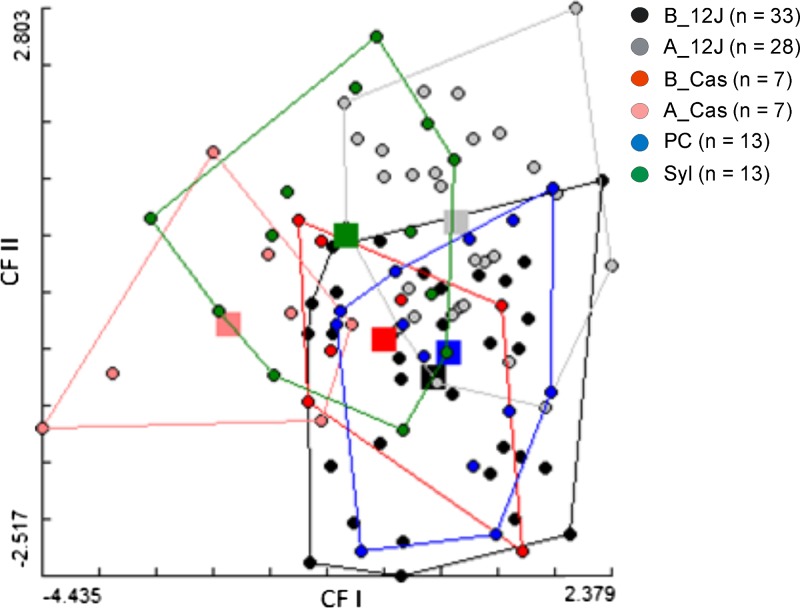
Scatterplots of discriminant analysis showing the variation in wing shape of females of *T. infestans* collected during prespraying (B) and postspraying (A) interventions in housing locations of 12 de Junio (12J), Casuarina (Cas), Puerto Casado (PC), and those captured in sylvatic environments (Syl) in the Paraguayan Chaco. The polygons enclose the individuals of each group. The centroids of each population are represented by squares and the ball inside represents each individual. This figure appears in color at www.ajtmh.org.

Wing shapes of females *T. infestans* collected at pre- (B) and postspraying (A) in the villages studied as well as those captured in sylvatic environments (Syl) in the Paraguayan Chaco, showed some significant differences as indicated by the discriminant analysis (Figure 4). The canonical factor 1 explained 40% of the variance, whereas the canonical factor 2 explained the 20% (60% of the total variation). Permutation test shows that the populations exhibited significant differences in the Mahalanobis distances (*P* < 0.003; Table 1).

**Table 1:** Mahalanobis distances in right wings of females of *Triatoma infestans* from Paraguayan Chaco and number of wings per population (*N* = 101)

Locality	Mahalanobis distances							
	Spraying period	Code	B_12J	R_12J	B_Cas	R_Cas	PC	Syl
12 de Junio	Prespraying (*N* = 33)	B_12J	0.00					
	Postspraying (*N* = 28)	A_12J	1.53*	0.00				
Casuarina	Prespraying (*N* = 7)	B_Cas	1.74	2.09	0.00			
	Postspraying (*N* = 7)	A_Cas	2.66*	2.95	2.78	0.00		
Puerto Casado	Prespraying (*N* = 13)	PC	1.35	1.62	1.91	2.87	0.00	
Sylvatic	na (*N* = 13)	Syl	1.83*	1.57	1.72	2.16	2.04	0.00

na = not apply. Specimens of *T. infestans* captured before (B) and after (A) pyrethroid spraying at 12 de Junio (12J), Casuarina (Cas) and Puerto Casado (PC) localities from Paraguayan Chaco. Wings of bugs captured in sylvatic environment (Syl) were included.

* Distances were significant at *P* < 0.0033 after Bonferroni correction.

The cross-checked classification of pre- and postspraying specimens from 12 de Junio showed that 42–46% were correctly reclassified; similar values were registered for pre- and postspraying specimens from Casuarina (42% for both). The lowest values registered were 23% and 30% from sylvatic and prespraying Puerto Casado specimens, respectively.

**Figure 5.**
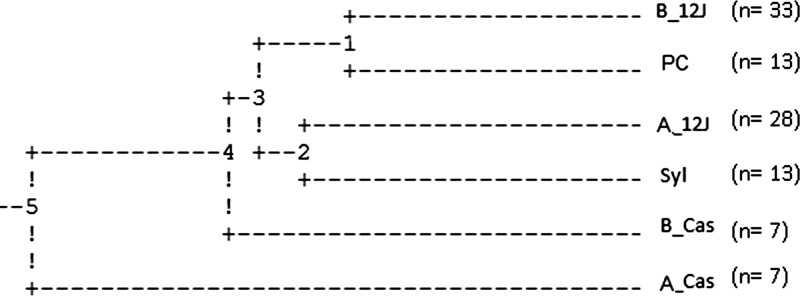
Unweighted pair-grouped method with arithmetic average dendrogram derived from Mahalanobis distances between specimens of *T. infestans* captured during prespraying (B) and postspraying (A) with pyrethroid at 12 de Junio (12J), Casuarina (Cas), and Puerto Casado (PC) localities from Paraguayan Chaco. Wings of bugs captured in sylvatic environment (Syl) were included. Numbers in brackets show the sample size for each population.

The UPGMA derived from Mahalanobis distances showed a closer proximity between the prespraying populations from 12 de Junio (B_12J) and Puerto Casado (PC). On the other hand, the postspraying population from 12 de Junio (A_12J) was more similar to the, geographically close, sylvatic population (Syl). The population from Casuarina (B_Cas) was clustered with the aforementioned, whereas the most different population was the postspraying Casuarina (A_Cas) (Figure 5).

A multivariate regression analysis did not show a significant effect of size on the shape (Wilks' Lambda = 0.5201, Approx. *F* = 1.13, df1 = 50, df2 = 368, *P* = 0.2556).

When the postspraying individuals from 12 de Junio were entered one by one to a discriminant analysis, the most (53%) were assigned to the sylvatic group. Besides, 25% of the postspraying specimens were assigned to prespraying population. A low percentage (11%) of bugs was assigned to other reference groups, Casuarina and Puerto Casado, respectively.

### Blood meal sources.

A total of 30 sylvatic specimens out of 49, captured in the area between 12 de Junio and 10 Leguas, were tested. The specimens assayed were 12 females, 10 males, six nymphs V, one nymph IV, and one nymph III. Of the 30 intestinal contents analyzed, four samples were reactive to the antisera tested (13.3%), 22 samples were not reactive (73.3%), and four samples (13.3%) were “border-line reactive” (low reaction in the ELISA test, not enough to consider reactive). The distribution of blood meal sources of four reactive *T. infestans* was human 75%, and dog 25%, the “border-line reactive” samples (four *T. infestans*) were cat 25%, human 25%, and goat 75% (Table 2). The five sylvatic triatomines were reacted to four out of five different blood meal sources by ELISA test, and were collected in nest of *Tabara major* and dry branches of “quebracho blanco” and “palo santo.” Sylvatic triatomines had human blood as meal source although they were captured in sylvatic areas with a distance of more than 1.5 km from the nearest house of both nearby villages. The sylvatic females showed blood meals from several hosts but chicken blood was not detected in any of these specimens. In relation to intradomicile triatomines blood meals in both villages, in a total of 12 and nine individuals with intestinal content from 12 de Junio and 10 Leguas, respectively, the human meals were predominant in both villages (41.7% and 33.3%), chicken meals were more frequent in 12 de Junio (33.3% and 11.1%), whereas dog meals were predominant in 10 Leguas (16.7% and 22.2%), to goat the blood meals were 8% and 11.1%, respectively. One specimen was reactive to antisera to cat from 10 Leguas (11.1%).

**Table 2:** Blood meal source by enzyme-linked immunosorbent assay test according to place of capture and distance to the nearest house in the surrounding areas of 12 de Junio and 10 Leguas villages from Paraguayan Chaco

Nearest village	Triatomine stage	Distance to the near house (in meters)	Blood meal source		Capture site
			Reactive	Border-line reactive	
10 L	1 ♂	1,952	Human		Nest of *Tabara mayor* in fallen Palo Santo tree
10 L	1NV	274		Goat	Dry branches of fallen quebracho blanco
10 L	1 ♀	3,438	Dog	Human	Dry branches of fallen quebracho blanco
12 J	1 ♀	3,345	Human	Cat, Goat	Dry branch of quebracho blanco
12 J	1 ♀	1,593	Human	Goat	Fallen and dry Palo Santo tree

10 L = 10 Leguas village; 12 J = 12 de Junio village; NV = nymph V.

## Discussion

Our study seems to be the first to evaluate wings similarity of *T. infestans* from the neighboring wild environment using geometric morphometric tools and shows a remarkable morphometric wings similarity between sylvatic and domestic populations and has suggested potential evidences on active dispersal ability and migration of these sylvatic individuals. Our results revealed that when the intradomicile postspraying individuals from 12 de Junio were entered one by one to the discriminant analysis, most were assigned to the sylvatic group. An entomological survey was conducted in all dwellings of 12 de Junio and Casuarina, resulting in a significant *T. infestans* infestation. However, in the entomological monitoring of postspraying at 3, 6, 9, and 12 months, very low triatomine abundance per house was detected, mostly adults inside the dwellings, because in these villages peridomestic structures did not exist. With the support of a trained dog, a search of sylvatic areas around 12 de Junio was performed 2 years after the spraying, which allowed us to capture sylvatic individuals of *T. infestans* for the first time in the Paraguayan Chaco. These sylvatic triatomines were genetically similar to those found in domiciles, as well as those captured in the monitoring postspraying.^12^^,^^13^ Unlike what was observed by us, previous studies in Argentina and the eastern region of Paraguay have attributed the reinfestation by *T. infestans* after spraying to residual foci in peridomestic structures.^18^^,^^26^^,^^39^ However, in the absence of peridomestic structures in the localities of our study area, with the exception of three dwellings in Casuarina, triatomines collected during postspraying periods may have been residual, sylvatic, or brought by passive transportation.

Our morphometric studies show that wing shapes of triatomines from Puerto Casado, an original community of this ethnic group, situated about 400 km from 12 de Junio, did not differ statistically from those captured in 12 de Junio (pre- or postspraying). This finding could be attributed to the fact that this village had its origin in a surrounding population of Puerto Casado, Alto Paraguay as described by Fabre (2005).^40^ Passive transport could have happened 27 years ago when they moved from Puerto Casado to 12 Junio or by successive visits of relatives from their former locality, very common practices in this ethnic group. In our study, morphometric results also show that triatomines from 12 de Junio did not have statistically significant differences between individuals captured in the baseline survey from those captured during postspraying periods as well as from sylvatic ones, and cytb genetic analysis previously obtained from these sylvatic individuals showed some haplotypes that were not found in the baseline insects, but they were present in some postspraying individuals.^12^ Although we cannot confirm whether the sylvatic foci were preexisting or not to the wide-community spraying of the study area, *T. infestans* populations would have a potential displacement between domestic and sylvatic areas, caused by spraying, as suggested by Ceballos and others (2011).^14^

Morphometric studies of triatomine populations from La Rioja, Argentina, have shown the presence of mixed reinfestation in dwellings attributed to survivors and to peridomestic populations.^24^ Our results indicate that mixed triatomine population occurs as well, when the shape of wings of postspraying individuals from 12 de Junio are sufficiently similar to those from sylvatic ones and these postspraying individuals are also similar to those captured during the prespraying. When the sylvatic individuals were analyzed by discriminant analysis, similar results were observed due to the sylvatic individuals were assigned to the postspraying group from 12 de Junio (results not shown).

Morphometric similarity in wings and gene flow observed previously between triatomines from pre- and postspraying captures, as well as from sylvatic ones, leads us to think in a dynamic mixed exchange between populations as has been highlighted in studies of La Rioja,^24^ or with potential involvement of other phenomena such as basal repopulation by residual foci due to faulty spraying, or by a potential reemergence of resistance to insecticides in use.^41^ On the other hand, pre- and postspraying triatomines from Casuarina were morphologically similar between them but different from 12 de Junio. In regard to this result, we consider as a possible explanation that these localities belong to different ethnic groups with different customs, and Casuarina dwellings present better quality and lower triatomine abundance. This differentiation could also be attributed to the diversity of construction materials, variety of hosts, infestation before insecticide spraying, as explained elsewhere and were mentioned as explicative factors in independent studies.^24^^,^^42^ Furthermore, other reasons could be related to the geographical distance between these two villages of 15 km that could be showing a certain structuring caused by geographical distances,^20^^,^^43^ or by subsequent domiciliary sprayings,^21^ which allow the differentiation of their different backgrounds.

In our study area, the origin of the postspraying triatomines remain uncertain; these postspraying individuals could come from intradomiciliary populations settled in the sylvatic area after a previous massive spraying, and maintain sufficient mobility, flying, or walking, as mentioned elsewhere allowing them to be capture inside the dwellings.^44^^–^^47^ Evidence supporting this finding in our study is the presence of intestinal content positive to human blood in adult triatomines captured between 1.5 and 3.4 km from the nearest dwelling and a fifth instar nymph fed on goat captured at 274 m of the nearest dwelling as well. The flight dispersal capacity of *T. infestans* range between 200 and 2,000 m would explain the result mentioned earlier.^48^^,^^49^ Furthermore, independently of the origin of sylvatic triatomines, different researchers have given great importance to the findings of sylvatic individuals in different countries and the impact that this evidence may have on control programs.^4^^,^^6^^,^^14^^,^^46^^,^^50^^–^^53^ In fact, this species generates a new challenge to the vector control programs because they can establish large colonies in nearby vegetation where chemical control is infeasible.

The low *T. infestans* densities observed could explain the absence of *T. cruzi* infection in these triatomines. Some studies have showed that low infection rates in domestic and peridomestic sites are associated with low *T. infestans* densities.^54^ On the other hand, control programs before and during surveillance phase have showed highly significant decrease of rate infection in domestic and peridomestic areas and recovery after blanket spraying could occur between 2 and 3 years.^54^^,^^55^ Our small sample of sylvatic triatomines was not infected with *T. cruzi* as well, and was captured with poor intestinal contents and mainly associated with goats or bird nets.^9^

Future studies should attempt to improve the understanding of the reinfestation process incorporating sylvatic populations, independently of the species, in the analysis of field studies. Genetic studies could shed light on the potential origin of preexisting sylvatic triatomines in the Chaco; supported by the ancestral knowledge of old indigenous people who permanently indicate that the triatomines have always came from the sylvatic area when the north wind blows. Therefore, additional studies should look for strategies that modify the ones currently used by the control program to eliminate triatomine populations surviving sprayings, and seek new surveillance tools to prevent extra domiciliary reinfestation pressure existing in these indigenous villages.
